# Editorial: Exploring modern approaches to address critical care challenges in risk stratification for cardiology

**DOI:** 10.3389/fcvm.2023.1326645

**Published:** 2023-11-15

**Authors:** Fardin Hamidi, Remo H. M. Furtado, Antonio Fagundes, Thomas A. Zelniker

**Affiliations:** ^1^ Division of Cardiology, Medical University of Vienna, Vienna, Austria; ^2^ Instituto do Coracao (InCor), Hospital das Clínicas HCFMUSP, Faculdade de Medicina, Universidade de São Paulo, São Paulo, Brazil; ^3^ Brazilian Clinical Research Institute, São Paulo, Brazil; ^4^ Division of Cardiology, D'Or Institute for Research and Education, Rio de Janeiro, Brazil

**Keywords:** cardiovascular care unit, critical care medicine, risk stratification, machine learning, risk factors, biomarkers

**Editorial on the Research Topic** Exploring Modern Approaches to Address Critical Care Challenges in Risk Stratification for Cardiology

The cardiovascular care unit plays a vital role in the broad field of critical care medicine. Originally known as the coronary care unit, this specialized medical unit was established in the 1940s to provide focused care for patients experiencing myocardial infarctions. However, its role expanded over time, transforming into the cardiovascular intensive care unit to accommodate the increasing complexity of cardiovascular illnesses, including acute exacerbations of heart failure and decompensated structural heart disease. Cardiovascular critical care patients represent a complex and heterogeneous group that has evolved over time, surpassing traditional risk estimation methods. Hence, there is a pressing need for innovative risk stratification approaches. Accurate risk stratification is paramount in determining the need for timely interventions or surgeries, guiding informed patient decision-making, enabling personalized therapy, and optimizing resource allocation (Figure 1) (1).

**Figure 1:**
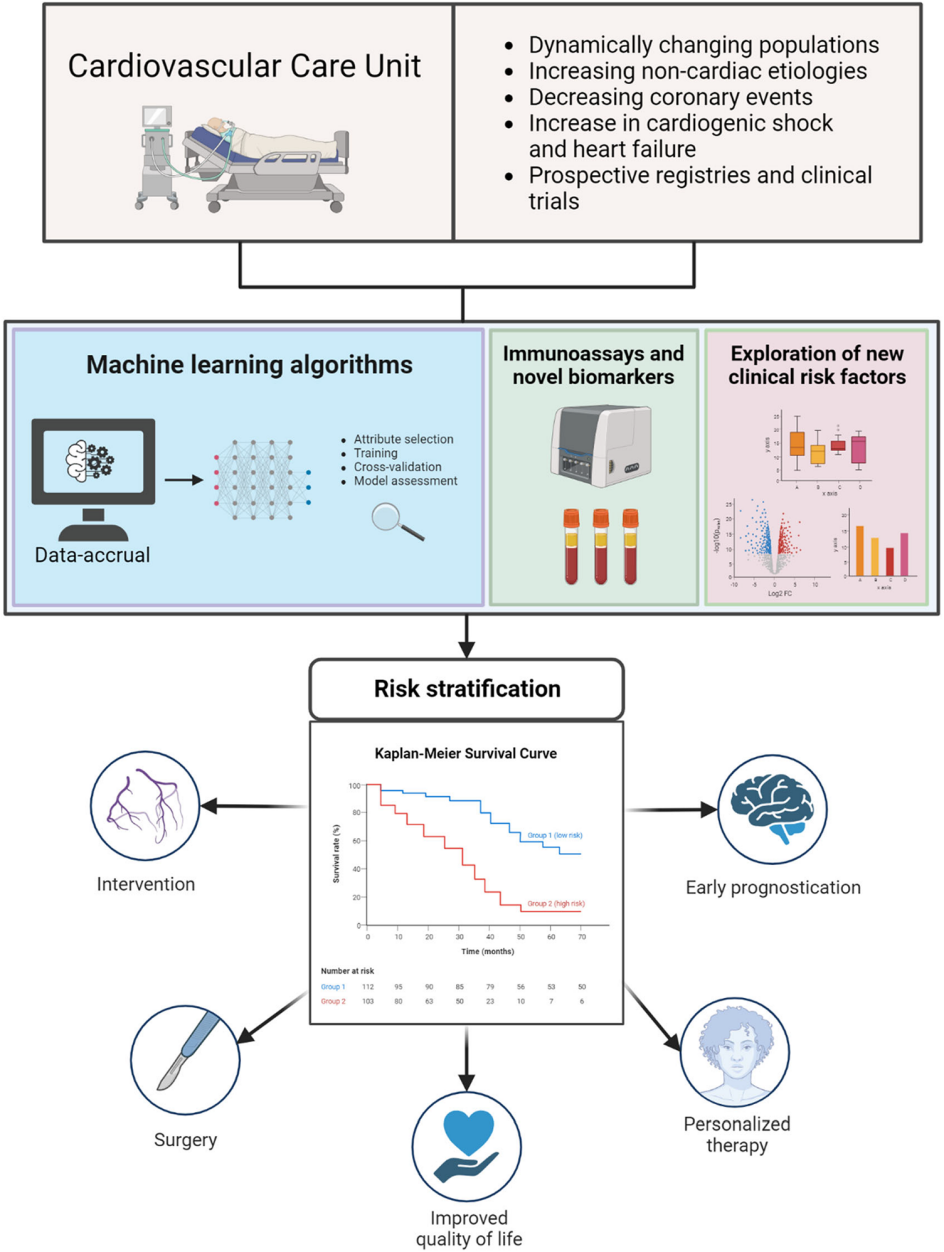
Evolving landscape of cardiovascular critical care and the need for accurate risk stratification leading to more effective and tailored treatment. Created with Biorender.com.

Such reasoning may drive studies like the one conducted by Yuan et al., a retrospective single-center study involving 2,179 patients diagnosed with type A and type B acute aortic dissection. In a comprehensive analysis, the authors explored factors predictive of in-hospital mortality and identified risk and protective factors. Additionally, they developed a risk score with considerable sensitivity and specificity for practical clinical use. This study is a valuable contribution to the field of risk stratification analyses in patients with acute aortic dissection and holds promise for improving the timely identification of surgical candidates in critical situations but will require external validation.

In another intriguing single-center prospective study, Jia et al. measured interleukin-33 (IL-33), soluble suppression of tumorigenesis-2 (sST2), myeloperoxidase (MPO), and matrix metalloproteinase (MMP)-9 levels in 155 patients with acute type A aortic dissection on admission. After a median follow-up of 6 months, patients with higher tertiles of sST2, MPO, and MMP-9 had higher rates of all-cause death. Additionally, these biomarker concentrations were found to be associated with greater aortic root diameter, maximum thoracic aorta diameter, and maximum pseudolumen area.

Amidst the rise of artificial intelligence and the advent of natural language models, the field of machine learning has piqued interest in predictive modeling across various medical fields. In a notable contribution, Chen et al. developed an eXtreme Gradient Boosting algorithm (XGBoost) to predict the risk of in-hospital mortality of patients hospitalized for heart failure. This comprehensive study encompassed a cohort of more than 20,000 patients, with training data originating from the Medical Information Mart for Intensive Care IV (MIMIC-IV) database and external validation carried out using the eICU Collaborative Research Database dataset (eICU-CRD). Moreover, the authors provide a freely accessible online calculator.

Another interesting retrospective study featured in this special issue explored the correlation between red blood cell distribution widths and both short-term and long-term mortality in patients with heart failure Ji and Ke. The clinical utility of red blood cell distribution width has traditionally been limited to hematological and oncological specialties. However, in recent years, red blood cell distribution has been increasingly recognized as a potential biomarker of heart failure progression due to its association with inflammatory anemia and sympathetic hyperactivity. In this retrospective analysis of 4,955 patients, higher red blood cell distribution width levels were strongly associated with an increased risk of death across the entire population even after robust co-variate adjustments.

In a retrospective observational study of 704 patients, Chen et al. explored the role of lactate/albumin ratio in patients with septic myocardial injury and found that higher quartiles of the lactate to albumin ratio were linked to increased hospital- and intensive care unit mortality following a linear pattern.

Machado et al. investigated the incidence and outcomes of sudden cardiac arrest prior to percutaneous coronary intervention (PCI) in patients with ST-elevation myocardial infarction (STEMI). Among 1,493 patients with STEMI, pre-PCI sudden cardiac arrest occurred in 133 (8.9%) patients and was associated with worse in-hospital outcomes, particularly when concomitant cardiogenic shock was present. Intriguingly, despite these adverse short-term outcomes, the longer-term mortality rates among patients who experienced pre-PCI sudden cardiac arrest were similar to those of patients without a history of sudden cardiac arrest.

Delirium is a strong and prevalent predictor of adverse outcomes in the intensive care setting. In a prospective single-center study, Kim et al. examined the relationship between nutritional indicators and delirium in 2,783 patients admitted to the cardiac intensive care unit of a tertiary hospital. This study suggests that serum albumin levels may outperform nutritional indices, including the Prognostic Nutritional Index and the Geriatric Nutritional Risk Index, for predicting delirium in the intensive care unit.

Overall, the studies featured in this special issue offer valuable insights into contemporary approaches to risk stratification, particularly in patients with acute aortic dissection, heart failure, and cardiac arrest. The future holds promise for more personalized treatment options and improved patient outcomes, driven by innovative technologies and a deeper understanding of complex risk factors.

## Disclosures

TAZ reports research grants from the Austrian Science Funds, the German Research Foundation, and Boehringer Ingelheim; honoraria for serving on advisory boards from Bayer AG and Boehringer Ingelheim; personal fees from Alkem Lab. Ltd, AstraZeneca, Bayer AG, Boehringer Ingelheim, and Sun Pharmaceutical Industries, and educational grants from Eli Lilly and Company. RHMF reports research grants and personal fees from AstraZeneca, Bayer, Servier, and Apsen; and research grants (received from his institution) from Pfizer, Libbs, Brazilian Ministry of Health, and University Health Network. AFJ reports educational grants from Boehringer Ingelheim, AstraZeneca, Novartis, and Merck Sharp & Dohme; honoraria for serving on advisory boards from Merck Sharp & Dohme.
